# Association Between Prenatal Exposure to Organochlorine Pesticides and Telomere Length in Neonatal Cord Blood

**DOI:** 10.3390/toxics12110769

**Published:** 2024-10-23

**Authors:** Ying Jiang, Ziyuan Xu, Meng Wang, Hongxiu Liu, Yuanyuan Li, Shunqing Xu

**Affiliations:** 1 Nanshan District Center for Disease Control and Prevention, Shenzhen 518054, China; 2 Key Laboratory of Environment and Health (HUST), Ministry of Education & Ministry of Environmental Protection, and State Key Laboratory of Environmental Health (Incubation), School of Public Health, Tongji Medical College, Huazhong University of Science and Technology, Wuhan 430030, China; d202281775@hust.edu.cn (Z.X.); lizongnan@126.com (M.W.); lhx@hust.edu.cm (H.L.); liyuanyuan@hust.edu.cn (Y.L.); 3 School of Environmental Science and Engineering, Hainan University, Haikou 570228, China

**Keywords:** organochlorine pesticides exposure, telomere length, prenatal exposure, newborns

## Abstract

**Objectives:** Environmental exposure may affect the telomere length (TL) of newborns, which is considered as an early biomarker indicating susceptibility for later life diseases. However, the effects of prenatal organochlorine pesticide (OCP) exposure on newborn TL remain unclear. This study aimed to investigate the association between prenatal exposure levels of OCPs during pregnancy and TL in neonatal cord blood. **Methods:** A total of 168 mother–infant pairs from a birth cohort in Wuhan, China, were included this study. The concentrations of hexachlorocyclohexanes (HCHs, including β-HCH, α-HCH, and γ-HCH), p,p’-dichlorodiphenyltrichloroethane (p,p’-DDT) and its metabolites (p,p’-dichlorodiphenyldichloroethane, p,p’-DDD; p,p’-dichlorodiphenyldichloroethylene, p,p’-DDE) were measured in cord blood. The associations between the OCPs and the TL in newborns were analyzed by a generalized linear regression model. Stratified analyses by newborn sex, maternal gestational weight gain, and pregnancy body mass index (BMI) were performed to evaluate if the associations were modified by these factors. **Results:** The detection rates of various OCPs ranged from 50.9% to 100.0%. The median concentration of p,p’-DDE was the highest (33.90 ng/g lipid), followed by β-HCH (8.67 ng/g lipid), and the median concentrations of the other OCPs were between 0.12 and 0.33 ng/g lipid. Among the all newborns, a two-fold increase in the γ-HCH concentration in the cord blood was significantly associated with a 0.024 (95% CI: −0.041, −0.007) decrease in the TL. After stratification by newborn sex, the inverse association between γ-HCH and the TL was only statistically significant in boys, but not in girls (*P* for interaction <0.05). In addition, after stratification by pre-pregnancy BMI, β-HCH and p,p’-DDT concentrations were significantly associated with a decreased TL in the overweight pre-pregnancy BMI group [−0.111 (95% CI: −0.203, −0.018) and −0.036 (95% CI: −0.049, −0.023), respectively]. **Conclusions:** Prenatal exposure to OCPs during pregnancy was associated with a decreased neonatal telomere length, which may be affected by the newborn sex and pre-pregnancy BMI. These findings may provide new insights into the mechanisms underlying OCP-induced adverse health effects.

## 1. Introduction

Organochlorine pesticides (OCPs) are persistent organic pollutants, mainly including hexachlorocyclohexane (HCH) and dichlorodiphenyltrichloroethane (DDT). Due to the toxic effects of OCPs on both humans and animals, the use of organochlorine pesticides has been banned for decades. However, OCPs have strong stability and anti-degradation ability in the environment, with a long half-life of several years. Therefore, they can still be widely detected in soil [1], surface water [2], air [3], and food [4], as well as in biological samples such as serum, adipose tissue [5], and breast milk [6], indicating widespread human exposure to OCPs. OCPs can enter the human body through a variety of exposure routes, including direct contact (skin), the respiratory tract, and the digestive tract, and they can accumulate in human bodies through food intake. Exposure to OCPs has been reported to be associated with a variety of adverse health outcomes in humans due to their immunotoxicity, reproductive developmental toxicity, endocrine toxicity, neurotoxicity, and carcinogenicity [7,8].

Until now, OCP exposure remains a widely concerning environmental health issue, especially the health risk of OCPs among vulnerable populations such as pregnant women and fetuses. Prenatal exposure to environmental pollutants can disrupt developmental processes, increase susceptibility to chronic diseases in later life, and lead to lifelong harm. OCPs have the ability to cross the placental barrier, and the high correlation coefficient between maternal and cord blood suggests that the levels of OCPs in cord blood can be used to estimate prenatal exposure levels [9]. In addition to daily exposure to OCPs, recent studies have confirmed that pregnant women mobilize OCPs in body fat during pregnancy, continuously exposing the fetus to OCPs [10].

Telomeres are repetitive, non-coding nucleotide sequences located at the end of chromosomes which can maintain genome integrity and chromosome stability [11]. As the telomere length gradually shortens with the increase of cell divisions, it serves as a biomarker of biological aging [12]. Telomere length has also been linked to many chronic diseases in adulthood, including cardiovascular disease [13], diabetes [14], various types of cancer, and other age-related diseases [15]. Telomeres are rich in guanine residues, which are highly sensitive to oxidative stress. OCPs can increase the oxidative stress level [16], which may affect the telomere length by reducing telomerase activity and inducing DNA damage [17,18,19]. The association between OCP exposure and the telomere length in adults has been reported, showing that OCPs may shorten the telomere length [20,21]. However, there is no study on the association between OCP exposure during pregnancy and the telomere length in newborns. A growing body of evidence underlines the importance of the newborn telomere length, which is regarded as an important factor in predicting telomere length in adulthood and is sensitive to environmental pollutants [22,23,24,25]. Therefore, exploring the potential effect of OCPs on the telomere length at birth may help to understand the fetal origin of disease in adulthood.

In the present study, we aimed to investigate the associations between the cord serum concentrations of OCPs [including HCH isomers (α-HCH, β-HCH, and γ-HCH) and p,p’-DDT and its metabolites (p,p’-dichlorodiphenyldichloroethane, p,’-DDD; p’-dichlorodiphenyldichloroethylene, p,p’-DDE)] and the newborn telomere length. We also analyzed the associations stratified by infant sex and maternal pre-pregnancy BMI, considering the lipophilic properties of the OCPs and the potential sex-specific effect.

## 2. Materials and Methods

### 2.1. Study Subjects

This study was based on a mother–infant birth cohort recruited at the Wuhan Women and Children Health Care Center, Wuhan, China. The criteria for inclusion in this birth cohort are as follows: (1) permanent residents of Wuhan; (2) completed the first prenatal care examination with singleton pregnancy less than gestational age of 16 weeks; (3) gave birth to a live newborn at the study hospital; (4) completed the questionnaire. Among the pregnant women who participated in the birth cohort between 2014 and 2015, 168 mother–newborn pairs who had cord blood samples available to determine OCPs and telomere length were selected in the analysis. All included subjects signed informed consents and agreed to collect biological samples. The research protocol was approved by the ethics committees of Tongji Medical College of Huazhong University of Science and Technology and Wuhan Women and Children’s Health Care Center.

### 2.2. Collection of Umbilical Cord Blood Samples

At delivery, neonatal cord blood was collected with a disposable syringe by trained obstetric nurses immediately after delivery. After standing at 4 °C for 2 h, the samples were centrifuged at 3000 rpm for 10 min to separate cord serum and cells, and then transferred into 2 mL polypropylene EP tubes. The samples were stored in a −80 °C refrigerator until OCP measurement and DNA extraction [26].

### 2.3. Determination of OCPs in Umbilical Cord Blood

The details about the sample pretreatment, determination of OCPs, and quality control were reported in our previous study [27]. We applied liquid–liquid extraction and gas chromatography–tandem mass spectrometry (GC–MS/MS) to determine nine organochlorine pesticides (α-HCH, β-HCH, γ-HCH, p,p’-DDD, p,p’-DDE, p,p’-DDT, o,p’-DDD, o,p’-DDE, and o,p’-DDT). The detection limit (limit of detection, LOD) was calculated as 3 times the signal-to-noise ratio. The method detection limits of α-HCH, β-HCH, γ-HCH, p,p’-DDD, p,p’-DDE, and p,p’-DDE were 0.3 pg/mL, p,p’-DDT was 1.1 pg/mL, p,p’-DDD was 1.2 pg/mL, and p,p’-DDT was 1.5 pg/mL.

The total serum lipids were determined gravimetrically. The lipid-adjusted concentrations of OCPs were calculated for analysis because OCPs are lipophilic.

### 2.4. Detection of Telomere Length in Umbilical Cord Blood Leukocytes

Telomere length in umbilical cord blood leukocytes was determined by the quantitative real-time polymerase chain reaction (qPCR) method as described previously [28]. The number of copies (telomere repeat copy number, T) of telomeric repeats of telomeric cells in cord blood leukocytes and single copy genes (single-copy gene copy number, S) were determined by real-time quantitative PCR, and the relative telomere length of cord blood genomic DNA was indicated by the T/S ratio [28]. Fifty randomly selected samples were mixed as the standard reference genomic DNA sample. For each sample, three replicates were set up for quality assurance, and the Ct value of the sample DNA is the average of the Ct values from the three replicates. The samples with a standard deviation of the Ct value of less than 0.3 were considered qualified.

### 2.5. Information Collection, Classification, and Definition of Covariates

The trained nurses conducted the face-to-face questionnaire to collect the basic information of the pregnant women, including age, education, occupation, living habits, and health status. Pregnancy-related information and current medical histories were collected through the hospital electronic medical record system, including last menstruation, time of delivery, labor time, pregnancy, pregnancy induced hypertension, gestational diabetes mellitus, baby sex, etc. Pre-pregnancy body mass index (PBMI) was calculated by dividing self-reported pre-pregnancy weight (kg) by the square of the measured height (m^2^). The women with different PBMIs were categorized into underweight (<18.5 kg/m^2^), normal (18.5–24 kg/m^2^), and overweight (>24 kg/m^2^), according to the definition for Chinese from the National Health Commission of the People’s Republic of China. There were no pregnant women reporting smoking and drinking in the study population. Passive smoking during pregnancy was defined as exposure of nonsmoking mothers to passive smoking during pregnancy (the father or other persons smoking in the household or workplace) [10].

### 2.6. Statistical Methods

The mean ± standard deviation (SD) and n (%) were expressed for continuous variables and categorical variables, respectively. The basic information collected in this study is very comprehensive and there was no missing information. The concentrations of OCPs lower than LOD were replaced by LOD/2, and then the concentrations corrected by total fat were log2 transformed for further analysis. The model results in our study are the regression coefficients and their 95% confidence intervals. A generalized linear regression model was used to analyze the association of exposure level of OCPs during pregnancy and cord blood telomere length. The inclusion of covariates adjusted based on the previous literature on the effects of pollutant exposure on telomere length and DDT exposure characteristics, or variable inflation factors (VIF), is used to test multicollinearity for each compound [29]. Highly collinear variables (VIF > 10) were omitted from further analysis (as shown in Table S1). We corrected the *p*-value (*P*_FDR_) by the false discovery rate (false discovery rate, FDR) to reduce the probability of false positives in multiple comparisons due to multiple OCPs being analyzed. The models were adjusted for maternal age (<25, 25–30, >30 years), pre-pregnancy BMI (<18.5, 18.4–24, >24 kg/m^2^), maternal education (12, >12 years), parity (1, >1), family economic situation (<100,000 yuan, ≥100,000 yuan), passive smoking during pregnancy (yes, no), and infant sex (male, female). Since previous studies have suggested a sex-specific relationship between prenatal exposure to OCPs and fetal growth [30,31,32], we performed a stratified analysis by infant sex (male, female) to explore whether infant sex modified the association between OCPs and newborn telomere length. In addition, the exposure level of OCPs may vary among pregnant women with different maternal ages [33] and pre-pregnancy BMIs [34], according to previous studies, so a stratified analysis was further performed to explore the effect of maternal age (<25, 25–30, >30 years) and pre-pregnancy BMI (<18.5, 18.4–24, >24 kg/m^2^) on the association between OCPs and neonatal cord blood telomeres. The RCS curve was performed and shown in Figure S1 to check the linear regression model. The interaction (*P* interaction) of these characteristics and OCP exposure level was assessed by Wald test (Wald test). All statistical analyses were carried out using SAS version 9.4 (SAS Institute, Inc., Cary, NC, USA). A two-sided *p*-value < 0.05 was considered statistically significant. Bonferroni correction for correcting *p*-value was applied.

## 3. Results

### 3.1. General Characteristics of the Study Subjects

The demographic characteristics of the participants are shown in Table 1. The mean age of the pregnant women was 28.2 ± 3.3 years, and the mean pre-pregnancy BMI was 20.8 ± 2.7 kg/m^2^. Among all the pregnant women, 93.5% of the pregnant women had more than 12 years of education, 91.0% were primiparas, 35.1% had an annual family income of more than 100,000 yuan, and 30.3% had passive smoking. The proportion of male infants (52.9%) was slightly higher than that of female infants (47.1%). The mean value of the neonatal cord blood telomere length (T/S) was 0.81 ± 0.4. In addition, the descriptive statistics for the different stratified groups indicated that passive smoking during pregnancy currently has a significant effect on telomere length differences.

### 3.2. Association of OCP Concentrations and Telomere Length in Neonatal Cord Blood

The association between the OCPs and the telomere length in neonatal cord blood is shown in Table 2. Among all the infants, only γ-HCH was significantly associated with a decreased telomere length in cord blood. After adjusting for the covariates, every 2-fold increase in cord blood of the concentration of γ-HCH was associated with a 0.024 (95% CI: −0.041, −0.007) decrease in the neonatal telomere length. No significant association was found between α-HCH, β-HCH, p,p‘-DDD, p,p’-DDE, and p,p’-DDT and the telomere length.

### 3.3. Stratified Analysis of the Association of OCPs and Telomere Length in Neonatal Cord Blood

The results of the stratified analysis of infant sex (male, female) are shown in Table 3. After adjusting for the covariates, for every 2-fold increase in the γ-HCH concentration in the male infants, the cord blood telomere length was shortened by 0.042 (95% CI: −0.070, 0.014). However, for every 2-fold increase in the concentration of α-HCH, p,p′-DDD, and p,p′-DDT in the female infants, the telomere length increased by 0.018 (95% CI: 0.002, 0.035), 0.017 (95% CI: 0.002, 0.032), and 0.031 (95% CI: 0.015, 0.047), respectively. The interaction effect of the infant sex and the concentration of γ-HCH on the telomere length in the neonatal cord blood was significant (*P*interaction < 0.05).

The results of the stratified analysis by maternal age (<25, 25–30, 30 years) are shown in Table 4. After adjusting for confounding factors, in the women aged <25 years, every 2-fold increase in α-HCH, p,p’-DDD, and p’-DDE was associated with a 0.025 (95% CI: −0.040, −0.011), −0.053 (95% CI: −0.0 96, −0.010), and 0.384 (95%CI: −0.701,0.067) decrease in the cord blood telomere length. In the women aged 25–30 years, for every 2-fold increase in γ-HCH concentration, the telomere length decreased by 0.025 (95%CI: −0.047, −0.003). However, no statistical association was found between the OCP concentrations and the telomere length in the pregnant women aged >30 years. The effect of maternal age and γ-HCH concentration during pregnancy on the telomere length in the neonatal umbilical cord blood may have a modifying effect (*P*interaction < 0.05).

The results of the stratified analysis by pre-pregnancy BMI (underweight, normal, overweight) are shown in Table 5. After adjusting for covariates, an inverse associations between γ-HCH and the telomere length was observed in the three pre-pregnancy BMI groups, but the association was only significant in the normal weight group (pre-pregnancy BMI 18.5–24 kg/m^2^), where every 2-fold increase in γ-HCH concentration was associated with a 0.027 (95% CI: −0.049, −0.006) decrease in the cord blood telomere length. In addition, in the overweight group (pre-pregnancy BMI > 24 kg/m^2^) group, per 2-fold increase in the concentration of β-HCH and p,p’-DDT, the cord blood telomere length decreased by 0.111 (95% CI: −0.203, −0.018) and 0.036 (95% CI: −0.049, −0.023), respectively. The interaction effects of the pre-pregnancy BMI and the concentration of γ-HCH and p,p’-DDT on the neonatal cord blood telomere length reached a significant level (*P*interaction < 0.05).

## 4. Discussion

To our knowledge, this is the first study reporting the association between OCP exposure during pregnancy and neonatal telomere length. This study found higher levels of γ-HCH were significantly associated with a decreased telomere length. After stratification by newborn sex, the significant association between γ-HCH and the TL was only observed in boys, but not in girls. After stratification by pre-pregnancy BMI, in addition to the inverse association between γ-HCH and the telomere length, β-HCH and p,p’-DDT concentrations were significantly associated with a decreased telomere length in the overweight pre-pregnancy BMI group.

Previous studies in adults have reported the associations between OCP exposure and telomere length. A cross-sectional study conducted in Tehran, Iran between 2016 and 2017 found a significant inverse association between serum concentrations of OCPs (including p,p’-DDD, p,p’-DDE, and p,p’-DDT; median: 15.48, 12.75, and 15.62 ng/g lipid, respectively) and leukocyte telomere length [20]. Another study from the United States found an increase in lifetime days of pesticide use for DDT was significantly associated with a decreased telomere length in male pesticide applicators [21]. Our findings were consistent with the reports indicating that OCP exposure may decrease telomere length. However, a study conducted in South Korea in 2006 found a positive correlation between p,p-DDE concentration (median: 334 ng/g lipid) and telomere length in adults [35]. The main reasons for the difference in the findings may due to the exposure period and concentration of OCPs, and the differences in the study design (longitudinal vs. cross-sectional) may also explain the inconsistent findings. Moreover, the mechanism for telomere length is not exactly the same as the changes in adult telomere length in early life. Given that the that rates of telomere attrition vary markedly at different ages, the loss of telomeric repeats in hematopoietic cells is a dynamic process that is deferentially regulated in young children and adults [36,37]; thus, the difference in research subjects (newborn, young adults, older adults) may also contribute to the discrepancies between studies. At present, the mechanism of action between the exposure to OCPs and telomere length is not clear. Based on the toxicological mechanism of OCPs in humans, we speculated that oxidative stress may be the potential mechanism of prenatal exposure to OCPs affecting the telomere length in newborns. Both animal experiments and population studies have found that exposure to OCPs increased levels of oxidative stress in the body [16,38]. Telomeres are rich in guanine residues, which are highly sensitive to oxidative stress and can affect the telomere length by reducing telomerase activity and inducing DNA damage [17,18].

A stratified analysis in our study showed the inverse association between prenatal exposure levels of OCPs and the neonatal telomere length was more pronounced in male infants. A marked disparity in antioxidant defense between males and females has been reported, and males have higher levels of plasma 15-F(2t)-isoprostane (oxidative stress markers) than females. On the other hand, we also found that prenatal exposure levels of OCPs were associated with longer newborn TLs in female infants, which may indicate the presence of a potential compensatory mechanism in response to OCP exposure insults. Compared with males fetuses, female fetuses have higher levels of estrogen [39], which plays a role in regulating superoxide dismutase and glutathione peroxidase gene expression, which can induce higher antioxidant enzyme levels in females [40]; thus, anti-oxidative mechanisms in females may mask the effects of the OCPs. Therefore, we speculated that these endocrine effects may lead to a sex-specific association between the exposure to OCPs and the neonatal telomere length during pregnancy. In this study, the statistical results were observed in the 25–30 year-old and the normal pBMI population, which is probably due to the large number of people in this group and the small sample size of the other subgroups. Maternal age has always been a risk factor for adverse birth outcomes, all of which are associated with the shortening of telomere length [41,42], and whether it may interact with OCP exposure on adverse health effects remains to be clarified in future studies. Due to China’s one-child policy during the sample collection period, most of the participants in the present research were primiparous. However, studies have shown that parity may affect exposure levels [43,44], so future research can explore the modifying effect of parity on the health of the offspring under environmental exposure. In addition, OCPs are fat-soluble, so it is possible that the risk may be observed in obese people, and it is necessary to expand sample validation in the future.

## 5. Conclusions

The results of this study indicate that prenatal exposure to OCPs may decrease telomere length in neonatal cord blood. The inverse association between OCPs and telomere length was stronger in male newborns. Telomere length in early life predicts telomere length in adulthood, while initial telomere length in newborns depends not only on genetic factors but also on various environmental factors. Therefore, avoiding environmental pollutants during pregnancy can effectively promote the health status of the newborn child and their future health.

## Figures and Tables

**Table 1:** Demographic characteristics of the participants (n = 168 mother–child pairs).

	Overall	Male	Female	Telomere Length	
Characteristics	*N* (%) or Mean ± SD	*N* (%) or Mean ± SD	*N* (%) or Mean ± SD	Mean ± SD (T/S ratio)	*p-Value* ^a^
**Total**	168 (100)	89 (53.0)	79 (47.0)	0.81 ± 0.40	
**Maternal age (years)**	28.2 ± 3.27	28.2 ± 3.50	28.2 ± 3.03	—	
<25	13 (7.74)	8 (8.99)	5 (6.33)	0.65 ± 0.22	0.49
25–30	108 (64.3)	55 (61.8)	53 (67.1)	0.82 ± 0.40	
≥30	47 (28.0)	26 (29.2)	21 (26.6)	0.82 ± 0.42	
**Pre-pregnancy BMI (kg/m^2^)**	20.8 ± 2.74	20.7 ± 2.71	20.9 ± 2.79	—	
<18.5	31 (18.5)	17 (19.1)	14 (17.7)	0.81 ±0.43	0.97
18.5–24	118 (70.2)	62 (69.7)	56 (70.9)	0.82 ± 0.41	
≥24	19 (11.3)	10 (11.2)	9 (11.4)	0.75 ± 0.25	
**Maternal education level**	—	—	—	—	
<Junior high school (<12 years)	11 (6.55)	5 (5.62)	6 (7.59)	0.75 ± 0.20	0.78
≥High school (≥12 years)	157 (93.5)	84 (94.4)	73 (92.4)	0.81 ± 0.41	
**Parity**	—	—	—	—	
1	153 (91.1)	80 (89.9)	73 (92.4)	0.79 ± 0.38	0.10
≥2	15 (8.93)	9 (10.1)	6 (7.59)	0.97 ± 0.53	
**Annual family income (RMB)**	—	—	—	—	
<100,000	109 (64.9)	61 (68.5)	48 (60.8)	0.81 ± 0.41	0.75
≥100,000	59 (35.1)	28 (31.5)	31 (39.2)	0.81 ± 0.39	
**Passive smoking during pregnancy**	—	—	—	—	
Yes	51 (30.4)	31 (34.8)	20 (25.3)	0.71 ± 0.32	**0.03**
No	117 (69.6)	58 (65.2)	59 (74.7)	0.85 ± 0.42	
**Gestation age at delivery (weeks)**	39.0 ± 1.06	40.0 ± 1.07	39.1 ± 1.06	—	
<37 (preterm birth)	1 (0.60)	0 (0.00)	1 (1.27)	0.70 ± -	— ^b^
≥37	167 (99.4)	89 (100)	78 (98.7)	0.81 ± 0.40	
**Child’s birth weight (g)**	3357 ± 402	3375 ± 374	3338 ± 435	—	
<2500 (low birth weight)	2 (1.19)	1 (1.12)	1 (1.27)	0.66 ± 0.05	— ^b^
≥2500	166 (98.8)	88 (98.9)	78 (98.7)	0.81 ± 0.40	

Note: BMI, body mass index; SD, standard deviation; a, telomere length was compared with nonparametric test (Wilcoxon or Kruskal–Wallis). b, comparisons were not tested due to few cases of preterm birth and low birth weight.

**Table 2:** Associations between cord serum OCPs and neonatal telomere length without preterm birth and low birth weight (*n* = 166).

OCPs (ng/g Lipid)	Model A β (95% CI)	*P_FDR_*	Model B β (95% CI)	*P_FDR_*
α-HCH	0.014 (−0.002, 0.031)	0.23	0.010 (−0.006, 0.026)	0.09
β-HCH	0.022 (−0.024, 0.067)	0.44	0.023 (−0.020, 0.066)	0.30
γ-HCH	**−0.015** (−0.033, −0.002) *	0.01	**−0.024** (−0.041, −0.007) *	0.001
p,p′-DDD	0.008 (−0.007, 0.023)	0.68	0.003 (−0.011, 0.018)	0.11
p,p′-DDE	0.028 (−0.004, 0.061)	0.12	0.030 (−0.001, 0.061)	0.08
p,p′-DDT	0.017 (−0.001, 0.033)	0.71	0.014 (−0.002, 0.031)	0.08

Note: A generalized linear regression model was used to analyze the association, and the concentrations of OCPs were corrected for total fat and log2 transformed. Model A did not adjust for confounding factors; Model B adjusted for maternal age, pre-pregnancy BMI, maternal literacy, parity, annual household income, passive smoking during pregnancy, and infant sex; * *p* < 0.05.

**Table 3:** Stratified analysis of OCP concentration and neonatal telomere length during pregnancy (infant sex).

OCP	Male Infant (*n* = 89) β (95% CI)	Female Infant (*n* = 79) β (95% CI)	*P*interaction
α-HCH	0.006 (−0.020,0.033)	**0.018** (0.002,0.035) *	0.44
β-HCH	0.029 (−0.034,0.091)	0.025 (−0.032,0.083)	0.86
γ-HCH	**−0.042** (−0.070, −0.014) *	−0.002 (−0.021,0.017)	**0.001**
p,p′-DDD	−0.016 (−0.040,0.008)	**0.017** (0.002,0.032) *	0.49
p,p′-DDE	0.032 (−0.015,0.079)	0.031 (−0.007,0.069)	0.15
p,p′-DDT	0.0003 (−0.029,0.029)	**0.031** (0.015, 0.047) *	0.11

Note: All models were corrected for maternal age, maternal education, pre-pregnancy BMI, average annual family income, maternal education, parity, and passive smoking during pregnancy. * *p* < 0.05.

**Table 4:** Stratified analysis of OCP concentration and neonatal telomere length during pregnancy (stratified by maternal age).

OCPs	Maternal Age Was <25 Years (*n* = 13) β (95% CI)	Maternal Age Was 25–30 Years Old (*n* = 108) β (95% CI)	Maternal Age Was >30 Years Old (*n* = 47) β (95% CI)	*P*interaction
α-HCH	**−0.025** (−0.040, −0.011) *	0.013 (−0.008, 0.033)	0.008 (−0.022, 0.037)	0.54
β-HCH	0.187 (−0.101, 0.273)	0.013 (−0.044, 0.069)	0.028 (−0.038, 0.094)	0.47
γ-HCH	−0.012 (−0.067, 0.044)	**−0.025** (−0.047, −0.003) *	−0.014 (−0.047, 0.018)	**0.002**
p,p′-DDD	**−0.053** (−0.096, −0.010) *	−0.007 (−0.028, 0.015)	0.02 (−0.001, 0.041)	0.65
p,p′-DDE	**−0.384** (−0.701, 0.067) *	0.028 (−0.013, 0.069)	0.027 (−0.024, 0.077)	0.18
p,p′-DDT	−0.001 (−0.046, 0.044)	0.01 (−0.012, 0.033)	0.034 (−0.009, 0.059)	0.14

Note: All models were corrected for maternal education, pre-pregnancy BMI, annual family income, maternal education, parity, passive smoking during pregnancy, and infant gender. * *p* < 0.05.

**Table 5:** Stratified analysis of the OCP concentrations and neonatal telomere length during prenancy (stratified by pre-pregnancy BMI).

OCP	Thinnish (Pre-Pregnancy BMI < 18.5 kg/m^2^) (*n* = 31) β (95% CI)	Normal (Pre-Pregnancy BMI 18.5–24 kg/m^2^) (*n* = 118) β (95% CI)	Overload (Pre-Pregnancy BMI of >24 kg/m^2^) (*n* = 19) *P*interaction β (95% CI)	
α-HCH	0.014 (−0.002, 0.031)	0.012 (−0.007, 0.032)	−0.010 (−0.035, 0.014)	0.54
β-HCH	0.022 (−0.024, 0.068)	0.021 (−0.032, 0.073)	**−0.111** (−0.203, −0.018) *	0.71
γ-HCH	−0.015 (−0.033, 0.002)	**−0.027** (−0.049, −0.006) *	−0.016 (−0.044, 0.013)	**0.004**
p,p′-DDD	0.008 (−0.007, 0.023)	0.009 (−0.009, 0.027)	0.02 (−0.001, 0.041)	0.77
p,p′-DDE	−0.029 (−0.004, 0.062)	0.029 (−0.0101, 0.067)	−0.025 (−0.52, 0.001)	0.26
p,p′-DDT	0.017 (−0.001, 0.034)	0.029 (−0.008, 0.050)	**−0.036** (−0.049, −0.023) *	**0.04**

Note: All models were corrected for maternal age, maternal education, parity, annual household income, passive smoking during pregnancy, and infant sex. * *p* < 0.05.

## Data Availability

The original data presented in this study are included in the article; further inquiries can be directed to the corresponding author.

## Supplementary Materials

The following supporting information can be downloaded at: https://www.mdpi.com/article/10.3390/toxics12110769/s1, Figure S1: Restrictive cubic spline regression model of testing the correlation between OCPs and telomere length; Table S1: Variance Inflation Factor (VIF) of Model B.
