# Supplementary material for: Association Between Prenatal Exposure to Organochlorine Pesticides and Telomere Length in Neonatal Cord Blood
Source: Toxics. 2024 Oct 23;12(11):769. doi: 10.3390/toxics12110769 (PMC11597908; doi:10.3390/toxics12110769)
Supplement: Supplementary file 1 [file toxics-12-00769-s001.zip › toxics-3223692-supplementary.pdf]

**Table S1. Variance Inflation Factor (VIF) of Model B.**

| **Compounds (ng/g lipid)** | Maternal age | Pre-pregnancy BMI | Maternal education level | Parity | Annual family income | Passive smoking during pregnancy | Infant's sex |
|---|---|---|---|---|---|---|---|
| **HCHs** | | | | | | | |
| α-HCH | | | | | | | |
| VIF | 1.0662 | 1.0686 | 1.1768 | 1.1767 | 1.0439 | 1.0690 | 1.0341 |
| β-HCH | | | | | | | |
| VIF | 1.0641 | 1.0566 | 1.1721 | 1.18615 | 1.0379 | 1.0693 | 1.0267 |
| γ-HCH | | | | | | | |
| VIF | 1.0852 | 1.0507 | 1.1934 | 1.17071 | 1.0335 | 1.0677 | 1.0266 |
| **DDTs** | | | | | | | |
| p,p′ -DDD | | | | | | | |
| VIF | 1.0641 | 1.0503 | 1.1726 | 1.17168 | 1.0340 | 1.0687 | 1.0286 |
| p,p′ -DDE | | | | | | | |
| VIF | 1.066 | 1.0518 | 1.1718 | 1.19326 | 1.0344 | 1.0796 | 1.0273 |
| p,p′ -DDT | | | | | | | |
| VIF | 1.0652 | 1.0509 | 1.1894 | 1.1700 | 1.0415 | 1.0688 | 1.0265 |

Note: before applying the model, variable inflation factors (VIF) is used to test multicollinearity for each compound. Highly collinear variables (VIF > 10) were omitted from further analysis

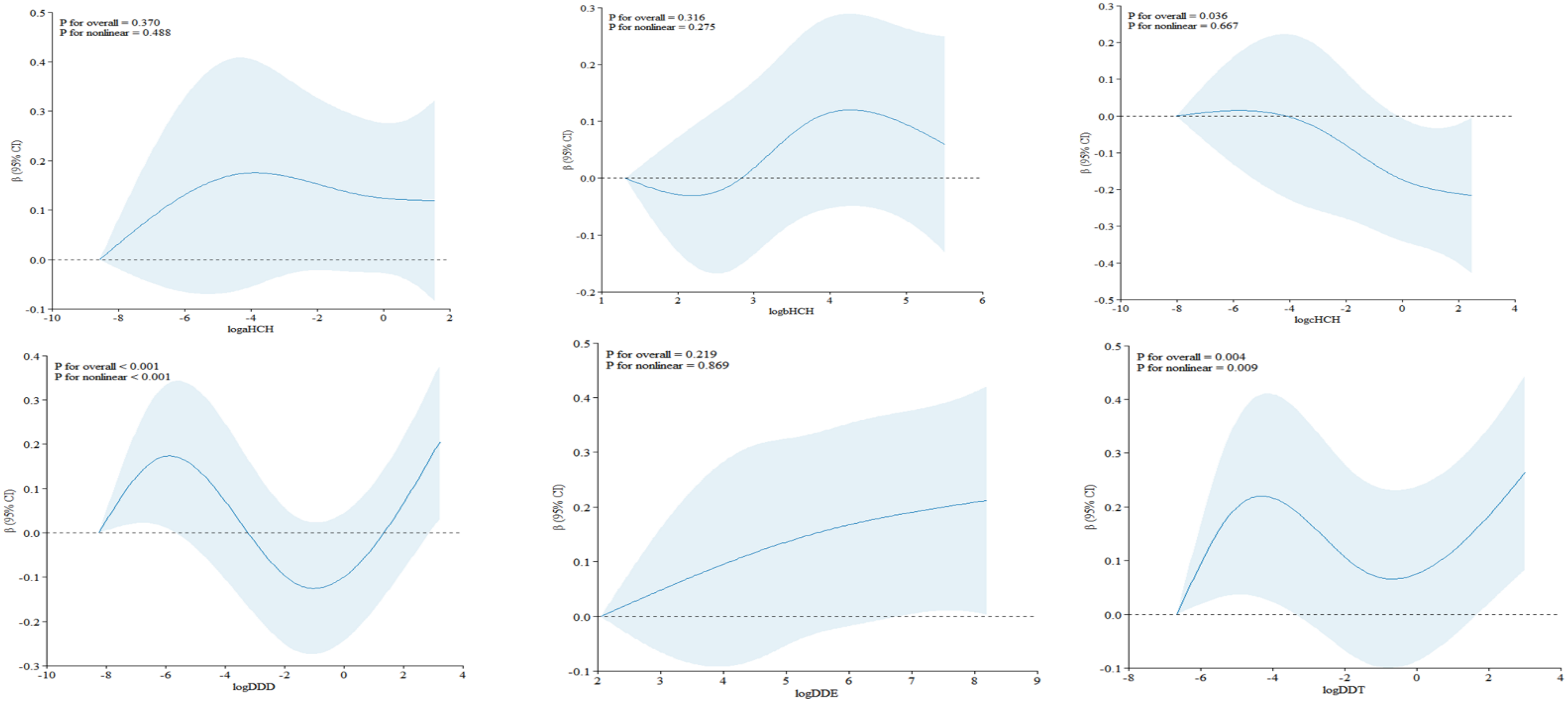


**Figure S1. Restrictive cubic spline regression model of testing the correlation between OCPs and telomere length**

Association between OCPs and telomere length using a restricted cubic spline regression model to check the linear regression model. Graphs show β for telomere length according to OCPs adjusted for maternal age maternal age, pre-pregnancy BMI, maternal education level, parity, annual household income, passive smoking during pregnancy, infant sex. The model was conducted with 4 knots at the 5th, 35th, 65th, 95th percentiles of OCPs (reference is the 5th percentile).Solid lines indicate β, and shadow shape indicate 95% CIs. CI, confidence interval.
